# Supplementary material for: Syndromic Surveillance Insights from a Symptom Assessment App Before and During COVID-19 Measures in Germany and the United Kingdom: Results From Repeated Cross-Sectional Analyses
Source: JMIR Mhealth Uhealth. 2020 Oct 9;8(10):e21364. doi: 10.2196/21364 (PMC7561445; doi:10.2196/21364)

Supplementary material

Table 1. Excluded symptoms from the analysis

	**Excluded symptoms**
**Creation in the model**	
	lump on the lower extremity
	pustule in the armpit
	indurated skin of the armpit
	indurated skin of the groin
	scarring in the groin
	scarring in the armpit
	pruritic armpit
	erythema of the armpit
	proctitis on examination
	recurrent candidiasis in females
	recurrent candidiasis in males
	ear abscess
	penile discharge foreskin
	hyphema
	raised bumps on the glans penis
	raised bumps on the penis shaft
	spot on the glans penis
	spot on the penis shaft
**Deletion from the model**	
	recurrent candidiasis
	severe acute respiratory syndrome associated coronavirus
**Modification in associated terms**	
	alopecia
	thinning hair
	spot on the penis

Table 2. Relative difference (log2 fold change values) in the proportions of Ada users' reported symptoms with statistically significant differences between the Baseline and COVID-19 Measures periods in Germany

ICD-10 groups & symptoms	Baseline (%)	Measures (%)	log2 fold change	P value[a]
**Cognition, perception, emotional state, and behaviour**				
diminished sense of taste [b]	.10	.48	2.26	<.001
hyposmia [b]	.05	.24	2.20	<.001
bad taste in mouth	.27	.41	.62	.002
depressed mood [b]	2.71	2.02	–.42	<.001
loss of motivation	.94	.59	–.67	<.001
inability to manage constant stress and demands at work [b]	.43	.21	–1.07	<.001
impaired concentration [b]	1.24	.91	–.46	<.001
dizziness	8.53	7.75	–.14	<.001
memory difficulty [b]	.57	.39	–.56	<.001
teeth grinding	.09	.04	–1.26	.033
aggressive behavior	.36	.25	–.51	.046
**Nervous system**				
excessive daytime sleepiness [b]	2.74	1.9	–.53	<.001
**General symptoms and signs**				
lowered temperature of the foot [b]	.34	.55	.71	<.001
body temperature decreased	.06	.14	1.12	.005
malaise [b]	2.53	1.25	–1.02	<.001
fatigue [b]	9.24	7.35	–.33	<.001
chills [b]	3.88	2.78	–.48	<.001
fever	6.32	4.95	–.35	<.001
headache	19.90	17.90	–.16	<.001
reduced performance	1.96	1.51	–.37	<.001
abulia	.66	.45	–.56	<.001
tenderness head	1.24	.97	–.36	.001
hyperhidrosis generalized	1.98	1.70	–.23	.015
**Musculoskeletal system**				
mark from bug bite	.04	.17	2.12	<.001
myalgia [b]	4.11	2.52	–.71	<.001
muscle cramps in the lower extremity	.38	.26	–.57	.014
feeling of heavy limbs	.11	.05	–1.10	.029
feeling of unstable knee	.08	.04	–1.25	.047
**Digestive system and abdomen**				
odynophagia [b]	3.99	2.33	–.77	<.001
vomiting [b]	2.62	1.83	–.51	<.001
nausea [b]	10.90	10.00	–.13	<.001
diarrhea [b]	5.90	5.37	–.14	.007

**Speech and voice**				
hoarseness [b]	.77	.36	–1.10	<.001
odynophonia [b]	.37	.24	–.64	.004
aphonia [b]	.06	.02	–1.64	.047
**Eye and adnexa**				
otalgia [b]	2.93	1.93	–.61	<.001
inability to clear the ear with changing barometric pressure [b]	.52	.31	–.77	<.001
aural fullness [b]	.85	.60	–.51	<.001
post orbital pain	1.92	1.59	–.27	.003
hearing deficit	.47	.34	–.49	.017
ocular discharge	.15	.08	–.87	.046
**Circulatory and respiratory systems**				
dyspnea [b]	3.67	5.38	.55	<.001
sneezing	.69	1.31	.93	<.001
chest pain [b]	7.11	8.34	.23	<.001
tragal tenderness	.05	.19	1.84	<.001
throat tightness	1.11	1.47	.40	<.001
sternal pain	.18	.28	.69	.009
dry throat	.59	.77	.39	.011
throat clearing [b]	.16	.26	.71	.011
nasal discharge [b]	7.20	4.75	–.60	<.001
sore throat [b]	8.44	6.14	–.46	<.001
cough [b]	12.90	10.20	–.34	<.001
sinus pain [b]	.71	.43	–.73	<.001
nasal congestion [b]	3.32	2.71	–.29	<.001
recurrent respiratory tract infections	.64	.39	–.73	<.001
**Skin and subcutaneous tissue**				
pruritic eyes [b]	.38	.81	1.10	<.001
dry skin of the hands [b]	.20	.46	1.21	<.001
skin rash of the hand	.27	.52	.97	<.001
skin rash of the upper extremity	.27	.46	.76	<.001
spot on the upper extremity [b]	.15	.30	.98	<.001
pruritic nasal cavity [b]	.16	.30	.94	<.001
pruritic hand	.13	.23	.90	.001
scaling skin of the intertriginous areas	.01	.05	2.51	.003
indurated skin of the foot	.00	.04	3.27	.011
skin rash of the antecubital fossa	.02	.07	1.61	.011
spot on the hand	.05	.11	1.11	.023
pruritic penis	.16	.08	–.92	.021
**Genitourinary system**				
breast tenderness	1.13	1.48	.39	<.001
pustule on the male genital	.07	.03	–1.40	.048

[a] Pearson chi-square test for differences between Baseline and Measures. *P* values were adjusted for multiple testing using the false discovery rate method.
[b] symptoms were also found to be significantly different between the Baseline and the Measures period in the United Kingdom

Table 3. Relative difference (log2 fold change values) in the proportions of Ada users' reported symptoms with statistically significant differences between the Baseline and COVID-19 Measures periods in the United Kingdom

ICD-10 groups & symptoms	Baseline (%)	Measures (%)	log2 fold change	*P* value[a]
**Cognition, perception, emotional state, and behaviour**				
hyposmia [b]	.07	.96	3.74	<.001
diminished sense of taste [b]	.09	.98	3.38	<.001
olfactory hallucination	.04	.11	1.49	.023
depressed mood [b]	4.11	2.49	–.72	<.001
anxiety	2.72	1.71	–.67	<.001
social phobia	.66	.31	–1.08	<.001
inability to manage constant stress and demands at work [b]	.35	.13	–1.43	<.001
impaired concentration [b]	1.71	1.18	–.53	<.001
memory difficulty [b]	.72	.43	–.73	<.001
previous depressive episode	.40	.21	–.94	<.001
suicidal tendencies	.79	.52	–.61	<.001
crying	.77	.52	–.57	.001
mood lability	1.39	1.08	–.35	.009
excessive worry	.51	.33	–.61	.010
extreme fear of situations with difficulty to escape or to get help	.16	.07	–1.26	.010
**Nervous system**				
excessive daytime sleepiness [b]	2.10	1.57	–.43	<.001
poorly restorative sleep	.62	.43	–.53	.015
**General symptoms and signs**				
lowered temperature of the foot [b]	.37	.56	.60	.006
fatigue [b]	12.70	10.10	–.33	<.001
malaise [b]	3.54	2.31	–.61	<.001
chills [b]	4.21	3.36	–.33	<.001
binge eating	.46	.24	–.91	<.001
cervical lymphadenopathy	1.25	.92	–.44	.001
weight gain	.66	.43	–.61	.002
irritability pediatric patient	.17	.07	–1.26	.007
weight loss	.59	.42	–.51	.025
**Musculoskeletal system**				
generalized arthralgia	1.04	.75	–.48	.001
hip pain	1.18	.93	–.34	.027
imbalance	.32	.20	–.69	.037
myalgia [b]	3.86	3.42	–.17	.037
**Digestive system and abdomen**				

gingival swelling	.09	.18	.95	.049
vomiting [b]	3.11	2.15	–.53	<.001
odynophagia [b]	1.29	.75	–.78	<.001
nausea [b]	12.50	11.40	–.14	<.001
diarrhea [b]	5.81	5.10	–.19	.001
abdominal pain	18.10	17.00	–.09	.005
flatulence	1.69	1.38	–.30	.018
change in stool color	.64	0.47	–.45	.046
**Speech and voice**				
aphonia [b]	.26	.07	–1.87	<.001
odynophonia [b]	.47	.21	–1.17	<.001
hoarseness [b]	.37	.23	–.68	.020
**Eye and adnexa**				
inability to clear the ear with changing barometric pressure [b]	.37	.21	–.85	.002
aural fullness [b]	1.03	.77	–.42	.008
otalgia [b]	3.00	2.62	–.20	.040
**Circulatory and respiratory systems**				
chest pain [b]	7.85	9.59	.29	<.001
pruritic throat	.49	.74	.59	.001
dyspnea [b]	5.71	6.45	.18	.001
throat clearing [b]	.15	.28	.90	.006
nasal pain	.41	.58	.49	.036
muscular tenseness of the chest muscles	.17	.28	.72	.047
sore throat [b]	10.50	7.20	–.55	<.001
nasal discharge [b]	5.63	3.31	–.77	<.001
nasal congestion [b]	5.16	3.61	–.51	<.001
sinus pain [b]	2.66	2.14	–.31	<.001
cough [b]	13.60	12.60	–.11	.005
tonsillopharyngeal exudate	.46	.31	–.59	.023
tonsillar enlargement	.43	.28	–.61	.026
**Skin and subcutaneous tissue**				
pruritic eyes [b]	.44	.89	1.02	<.001
pruritic nasal cavity [b]	.10	.28	1.52	<.001
urticaria	.18	.33	.86	.004
dry skin of the hands [b]	.14	.28	.96	.004
scaling skin of the face	.05	.13	1.38	.015
spot on the upper extremity [b]	.18	.30	.72	.037
scrotal pruritus	.16	.06	–1.27	.011
**Genitourinary system**				
vaginal pain	.62	.44	–.51	.019

[a] Pearson chi-square test for differences between Baseline and Measures. *P* values were adjusted for multiple testing using the false discovery rate method.

[b] symptoms were also found to be significantly different between the Baseline and the Measures period in Germany

Table 4. Relative difference (log2 fold change values) in the proportions of Ada users' reported symptoms with statistically significant differences between the same period as the Baseline and COVID-19 Measures periods in 2019 in Germany

**ICD-10 groups & symptoms**	**Baseline (%)**	**Measures (%)**	**log2 fold change**	***P* value[a]**
**General symptoms and signs**				
malaise	2.57	1.82	–.50	<.001
fatigue	8.97	7.98	–.17	.014
chills	4.01	2.61	–.62	<.001
fever	5.67	3.65	–.64	<.001
headache	21.1	17.6	–.26	<.001
**Musculoskeletal system**				
myalgia	4.03	2.76	–.55	<.001
**Circulatory and respiratory systems**				
nasal discharge	8.15	6.24	–.39	<.001
sore throat	8.53	7.12	–.26	<.001
cough	11.3	8.8	–.37	<.001
sinus pain	.84	.56	–.59	.029

[a] Pearson chi-square test for differences between Baseline and Measures. *P* values were adjusted for multiple testing using the false discovery rate method.

Table 5. Relative difference (log2 fold change values) in the proportions of Ada users' reported symptoms with statistically significant differences between the same period as the Baseline and COVID-19 Measures periods in 2019 in the United Kingdom

**ICD-10 groups & symptoms**	**Baseline (%)**	**Measures (%)**	**log2 fold change**	***P* value[a]**
**Genitourinary system**				
breast tenderness	2.59	1.85	–.49	<.001

[a] Pearson chi-square test for differences between Baseline and Measures. *P* values were adjusted for multiple testing using the false discovery rate method.

Table 6. Correlation between the significantly different symptoms in Germany and three weather variables (temperature, precipitation, and sunshine)

	Temperature		Precipitation		Sunshine	
**Symptoms**	ρ	***P* value**[a]	ρ	***P* value**[a]	ρ	***P* value**[a]
dyspnea	.08	.825	.02	.964	.25	.458
diminished sense of taste	–.46	.145	.05	.964	–.25	.458
pruritic eyes	.50	.116	–.37	.964	.76	.023
hyposmia	–.34	.301	–.04	.964	–.14	.700
chest pain	–.80	.007	.09	.964	–.67	.061
dry skin of the hands	–.51	.104	–.37	.964	–.09	.773
lowered temperature of the foot	–.86	.003	.09	.964	–.79	.023
spot on the upper extremity	.80	.007	–.09	.964	.75	.023
pruritic nasal cavity	.49	.116	–.49	.917	.77	.023
throat clearing	.03	.917	–.06	.964	.26	.458
nasal discharge	–.60	.052	–.02	.964	–.41	.243
odynophagia	–.68	.027	–.04	.964	–.60	.070
malaise	–.64	.040	.17	.964	–.59	.070
myalgia	–.74	.012	.05	.964	–.62	.070
sore throat	–.53	.104	.02	.964	–.33	.349
cough	–.75	.011	.06	.964	–.60	.070
fatigue	–.41	.196	.05	.964	–.28	.456
otalgia	–.73	.014	.08	.964	–.58	.071
chills	–.77	.010	.03	.964	–.64	.070
excessive daytime sleepiness	.17	.638	.23	.964	.05	.856
hoarseness	–.61	.052	.29	.964	–.58	.071
vomiting	–.64	.040	–.12	.964	–.65	.070
depressed mood	–.52	.104	.32	.964	–.61	.070
inability to manage constant stress and demands at work	–.20	.572	.50	.917	–.55	.092
sinus pain	–.76	.010	.15	.964	–.72	.033
nasal congestion	–.60	.052	–.19	.964	–.34	.349
inability to clear the ear with changing barometric pressure	–.44	.169	.23	.964	–.46	.181
impaired concentration	–.37	.256	.18	.964	–.42	.240
aural fullness	–.52	.104	.44	.964	–.60	.070
nausea	.05	.890	.28	.964	–.17	.627
memory difficulty	–.15	.669	.22	.964	–.27	.456
odynophonia	–.55	.088	.03	.964	–.34	.349
diarrhea	.66	.035	.15	.964	.49	.147
aphonia	–.23	.519	–.49	.917	–.10	.773

[a] Pearson chi-square test for differences between Baseline and Measures. *P* values were adjusted for multiple testing using the false discovery rate method.

Figure 1. Correlation between the significantly different symptoms in Germany and three weather variables (temperature, precipitation, and sunshine)

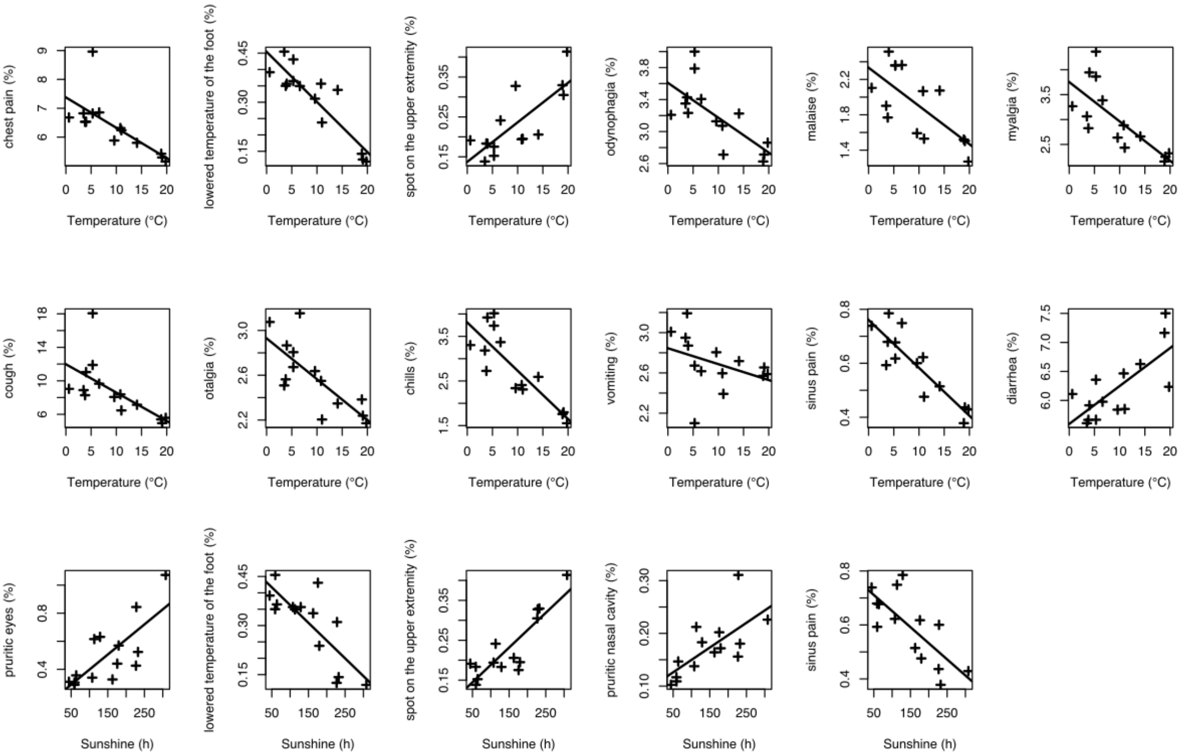

Supplement: Multimedia Appendix 1 [file mhealth_v8i10e21364_app1.pdf]
